# Deconstructing Adipose Tissue Heterogeneity One Cell at a Time

**DOI:** 10.3389/fendo.2022.847291

**Published:** 2022-03-25

**Authors:** Dylan J. Duerre, Andrea Galmozzi

**Affiliations:** ^1^ Department of Medicine, University of Wisconsin-Madison, School of Medicine and Public Health, Madison, WI, United States; ^2^ Department of Biomolecular Chemistry, University of Wisconsin-Madison, School of Medicine and Public Health, Madison, WI, United States; ^3^ University of Wisconsin Carbone Cancer Center, University of Wisconsin-Madison, School of Medicine and Public Health, Madison, WI, United States

**Keywords:** adipose tissue, white adipocytes, brown adipocytes, beige adipocytes, thermogenesis, tissue heterogeneity, obesity, type 2 diabetes

## Abstract

As a central coordinator of physiologic metabolism, adipose tissue has long been appreciated as a highly plastic organ that dynamically responds to environmental cues. Once thought of as a homogenous storage depot, recent advances have enabled deep characterizations of the underlying structure and composition of adipose tissue depots. As the obesity and metabolic disease epidemics continue to accelerate due to modern lifestyles and an aging population, elucidation of the underlying mechanisms that control adipose and systemic homeostasis are of critical importance. Within the past decade, the emergence of deep cell profiling at tissue- and, recently, single-cell level has furthered our understanding of the complex dynamics that contribute to tissue function and their implications in disease development. Although many paradigm-shifting findings may lie ahead, profound advances have been made to forward our understanding of the adipose tissue niche in both health and disease. Now widely accepted as a highly heterogenous organ with major roles in metabolic homeostasis, endocrine signaling, and immune function, the study of adipose tissue dynamics has reached a new frontier. In this review, we will provide a synthesis of the latest advances in adipose tissue biology made possible by the use of single-cell technologies, the impact of epigenetic mechanisms on adipose function, and suggest what next steps will further our understanding of the role that adipose tissue plays in systemic physiology.

## Introduction

Metabolic flexibility is a crucial trait of organisms strongly selected for across evolutionary time. As environmental conditions change, organisms must readily adapt physiologically and behaviorally to ensure survival. While nutrient availability fluctuates with time, organisms capable of storing excess energy under calorie abundance are more resilient to starvation and thermogenic stress during times of limited nutrient supply (1). Wired to be highly plastic and respond rapidly to changing cues, adipose tissue is an essential organ that serves multiple important functions, including storage and release of caloric substrates. However, the modern era fuels chronic abundance of calorically-dense foods and significantly contributes to the epidemic of metabolic dysfunction (2). Diseases such as obesity and type II diabetes (T2D) are amongst the most significant contributors to pathologic morbidity and mortality in developed nations and are wreaking havoc on quality of life. Dysfunctional adipose tissue is often one of the early indicators of metabolic disarray (3). To understand the mechanisms underlying metabolic diseases, a detailed understanding of adipose tissue in the healthy state, and the changes that occur during the onset of dysfunction, are pertinent areas of interest that are gaining significant attention over the last few decades (3–5). Initially described as a connective tissue capable of storing triglycerides, our modern understanding of adipose tissue demonstrates that it is highly heterogenous from both anatomic and physiologic perspectives. In addition to its role in energy storage and release, adipose depots are also major contributors to systemic physiology through endocrine signaling, regulation of the inflammatory state, and control of behavior. In particular, the past 5-10 years have seen rapid accelerations of resolution into adipose tissue dynamics. Implementation of modern techniques such as genetic engineering, lineage-tracing and single-cell characterizations revealed that adipose tissue is quite complex in composition and function. Resident cell types, including mature lipid-storing adipocytes, adipocyte precursor cells (APCs), and immune cells are major players that contribute to the overall function and remodeling. Heterogeneity subsists beyond the existence of divergent cell types, as individual cells exhibit unique characteristics indicative of distinct functional roles.

## Heterogeneity of White Adipocytes

White adipose tissue (WAT) represents most prominent adipose depots by weight and volume in mice and humans. WAT is generally regarded as the major storage site of nutritional metabolites upon excess intake in the form of lipids such as triglycerides (TGs). To safely harbor energy stores and avoid lipotoxic effects, white adipocytes maintain large, unilocular lipid droplets (LDs) that are capable of remodeling for expansion or contraction depending on the nutritional status. Integrating its lipid storage capacity to systemic homeostasis, WAT exerts endocrine functions *via* regulated release of adipokines such as leptin and adiponectin, cytokines and other lipid-derived signaling molecules (6, 7). The entire suite of adipokines and detailed descriptions of their endocrine effects are covered elsewhere (6–9), however it is important to provide adequate context of the major endocrine mediators. Leptin and adiponectin are the most prominent and best characterized adipokines and have major effects on systemic physiology. Leptin primarily communicates an adipose-derived nutritionally-fed status to the hypothalamus, in turn decreasing appetite and promoting energy expenditure (6, 10). Leptin also exerts effects on the periphery, including major metabolic organs such as the liver (11) and skeletal muscle (12, 13). Whereas leptin exerts most of its’ effects in the brain, adiponectin primarily acts in the periphery to modulate insulin sensitivity, glucose and fatty acid metabolism and inflammatory processes in major organs (8). Perturbations to homeostatic physiology such as obesity and age-related disease are strongly associated with altered circulating levels of both leptin and adiponectin (14–16). Therefore, the cellular composition of adipose tissue and the endocrine capacity of depots is likely to be remodeled in response to environmental and temporally-induced changes.

Of note, not all WAT depots function similarly and may not respond to environmental cues in the same manner. Subcutaneous WAT (sWAT) and inguinal WAT (iWAT) in humans and mice, respectively, are functionally distinct from visceral WAT (vWAT) and perigonadal/epididymal WAT (pg/eWAT) (17). Furthermore, the plasticity and responses of individual depots can vary widely depending on biological sex (18). The heterogeneity of WAT function arises from the diversity of cell types that reside in the adipose tissue, including the mature adipocytes themselves. New advances in analytic techniques are peeling back the layers of complexity that exist within the depots, and reveal that mature adipocytes have distinct characteristics and functions from one another.

**Figure 1:**
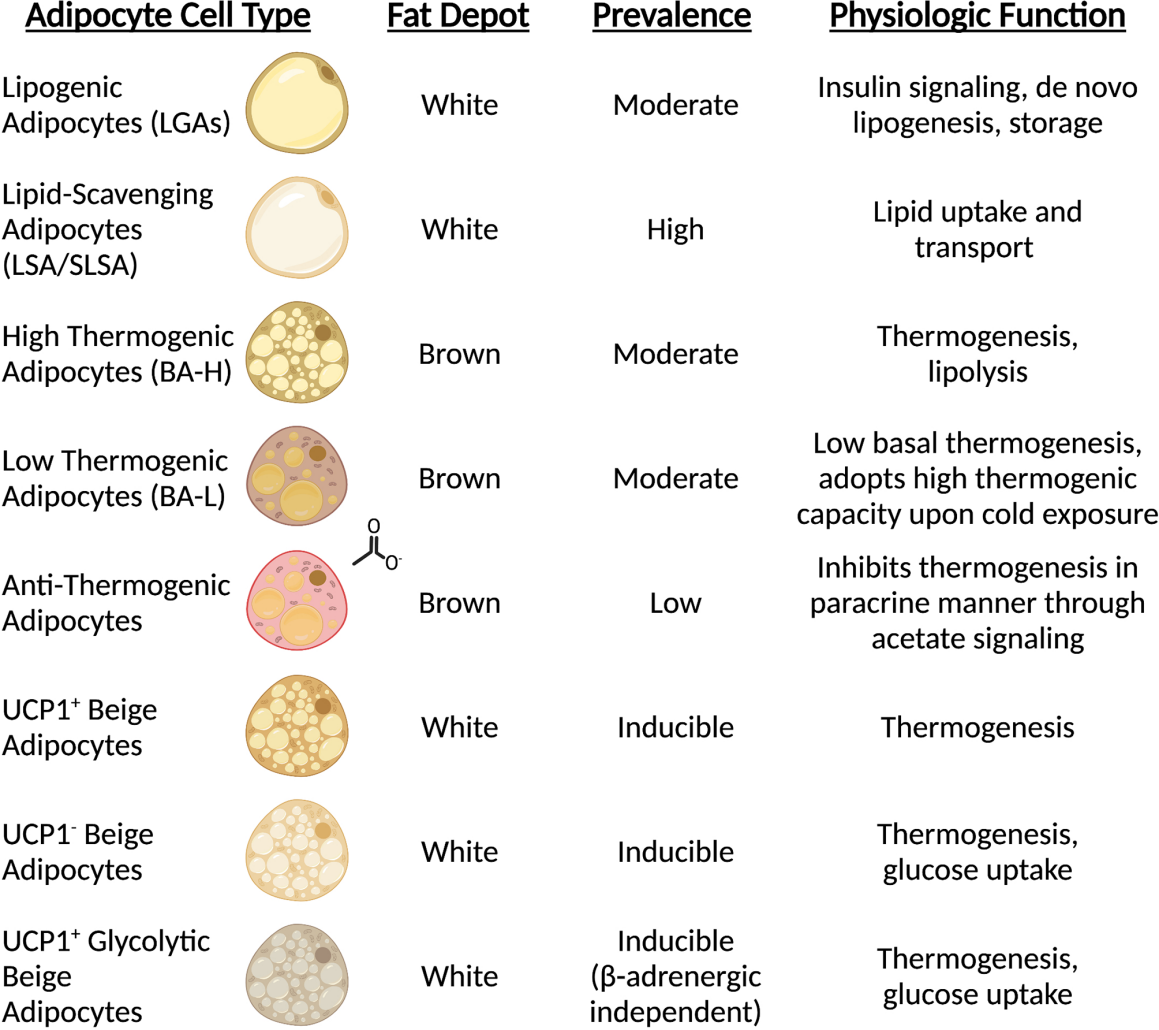
Mature adipocytes possess distinct spatial and functional roles. Sc-RNAseq revealed all adipocytes are not equal, and subpopulations carry out unique functions. The prominence of mature adipocyte cell types is dependent on type (white vs. brown) and anatomical position (subcutaneous vs. visceral vs. interscapular).

Until recently, the analysis of mature adipocytes has been technically difficult due to the extreme lipid contents which are largely incompatible with fluorescence activated cell sorting (FACS) and single-cell analysis strategies. However, single-nuclei RNA sequencing (sn-RNAseq) and spatial transcriptomics have created an avenue to explore the complexity of adipocyte status and function *in vivo*. While current knowledge keeps expanding, it is now clear that distinct adipocyte populations exist within WAT depots. Two independent studies characterizing the subpopulations of mouse and human WAT have described two predominant types of mature adipocytes. The first type includes insulin-sensitive and lipogenic adipocytes characterized by elevated levels of classical adipogenic markers such as *PPARG*, *PLIN* and *de novo* lipogenesis enzymes (referred to as LGAs/Adipo^PLIN^ populations) (19, 20). Instead, the second, distinct subpopulation is marked by high expression of genes involved in lipid uptake and handling (i.e., LSAs/Adipo^LEP^ populations), suggesting that this subtype of adipocytes relies on scavenging lipids rather than *de novo* lipid synthesis (19, 20) (**Figure 1**). Notably, both studies showed that this latter population of cells constitutes the largest fraction of mature adipocytes in both visceral and subcutaneous depots in adult mice and humans (19, 20). *In vivo* studies also indicate that this subpopulation is less responsive to insulin in humans when compared to the smaller lipogenic population (20). Recent work from the Granneman lab has confirmed the identification of these two subclasses during postnatal iWAT development in proportions as described previously (21). In line with their gene expression signatures, these two distinct populations may also have distinct adipokine secretion profiles. The Corvera lab also identified two types of adipocytes resembling those described above that appear to preferentially express adiponectin or leptin, respectively (22). Therefore, the endocrine effects of these fat cells may play distinct roles in coordinating physiologic responses. Future efforts aiming to functionally characterize these two populations and their contributions to tissue homeostasis will certainly be revealing. Although the majority of mature adipocytes appear to fall into the lipogenic or lipid-scavenging class, less abundant subtypes have also been described. Sárvári & Van Hauwaert et al. identified a population of adipocytes with a transcriptional profile resembling the lipid-scavenging subpopulation, but enriched in stress response genes (19). Instead, Bäckdahl & Franzén and coworkers reported a cluster of rare adipocytes that resemble traditional adipogenic population with enhanced expression of genes involved in retinol metabolism (20). Further investigation into these less abundant subtypes will shed light on their function and how they relate to the two major populations that dominate the fat depots.

One challenge that the field of adipose biology continuously encounters is deciphering differences in cellular composition based on species and adipose depot locations. To better understand where and under what conditions WAT depots differ from one another, a recent pre-print from the Rosen group describes an extensive single-cell characterization of visceral and subcutaneous WAT stratified by species (23). This adipose “single-cell atlas”, in addition to building on the characterization of adipocyte subpopulations, revealed that mouse adipocytes, while distinct from one another at the transcriptional level, do not clearly map to human adipocyte counterparts (23). Furthermore, mouse adipocytes do not show clear differences in subpopulation enrichment specifically in iWAT or eWAT in a healthy, lean state, contrary to depot-specific adipocyte enrichments found in human sWAT and vWAT (23). Future work will help characterize the inter- and intra-species differences in adipose depot composition. However, the evidence generated in humans and mice to date does not necessarily translate to one another, warranting caution when extrapolating data between species.

## Heterogeneity of Brown Adipocytes

**Figure 2:**
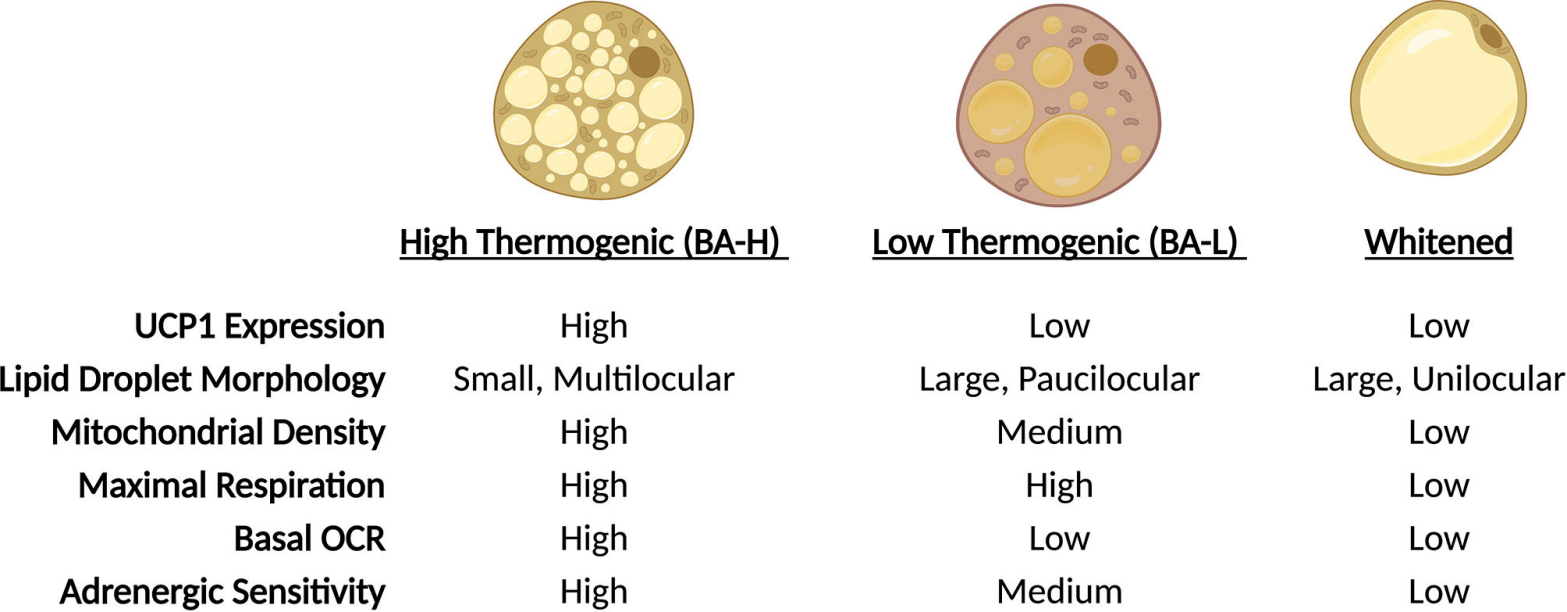
Brown adipocytes exhibit unique plasticity in response to environmental cues. In response to changes in ambient temperature, BAT-resident adipocytes undergo physiologic changes. Through trans-differentiation, adipocytes alter lipid droplet morphology, expression of thermogenic genes and mitochondrial function. During cold exposure, adipocyte proportions shift towards BA-H prominence, while thermoneutrality shifts the population towards a whitened state.

Brown adipose tissue (BAT) dissipates energy in the form of heat thus increasing energy expenditure and, simultaneously, putting a brake on excessive fat deposition (24). The discovery of BAT in adult humans and its correlation with body mass index (25–28), especially in older people (25), suggests a potential role of BAT in adult human metabolism and whole-body homeostasis. Similar to WAT, BAT consists of many different cell types, including immune cells, precursors and mature adipocytes, and the dynamics of resident cells reflect the adaptation to environmental changes, such as temperature, nutritional state and age. In response to prolonged cold exposure or direct β-adrenergic stimulation, BAT activates lipolytic and thermogenic machinery. The heat production of brown adipocytes is largely attributed to the expression of the uncoupling protein 1 (UCP1), a mitochondrial transmembrane protein that decouples the proton gradient generated by the electron transport chain from ATP synthesis, dissipating the energy as heat. However, other UCP-1 independent mechanisms that contribute to BAT thermogenesis, such as the futile creatine cycling, have been discovered (29–33) and is now clear that the thermogenic capacity of these cells extends beyond Ucp1-expression (31). As a matter of fact, elegant work from Cinti and colleagues in 2002 (34), subsequently confirmed by Spaethling & Sanchez-Alavez et al. (35), reported uneven expression of Ucp1 across brown adipocytes, providing a first peek of BAT heterogeneity. These observations were further supported by *in vivo* data using adipocyte lineage tracing reporter mice that confirmed the existence of two distinct subpopulations of brown adipocytes residing within interscapular BAT (iBAT), marked by high (BA-H) and low (BA-L) expression of adiponectin (36) (**Figure 1**). Further characterization revealed that *Ucp1* expression directly correlates with adiponectin levels, and that BA-L cells have decreased mitochondrial number, low basal oxygen consumption rate (OCR), and larger lipid droplets (36). Transcriptional profiling highlighted fatty acid uptake, rather than *de novo* synthesis, as the predominant metabolic process in BA-L subtype (36). Interestingly, the emergence of these two populations is not linked to sympathetic innervation as it might be expected, but may instead be due to differences in sensitivity to adrenergic stimulation. High-Adiponectin, high-Ucp1 cells (BA-H) also exhibit higher expression of *Adrb3* and are characterized by elevated basal and uncoupled OCR in response to norepinephrine compared to their BA-L counterparts (36). However, both BA-H and BA-L cells show similar maximal respiration, indicating that BA-L cells still possess elevated mitochondrial potential that can be activated if needed (36). Similarly, brown adipocytes can undergo a “whitening” effect upon thermoneutral housing, dampening thermogenic gene expression and acquiring a white adipocyte-like morphology and phenotype (37) (**Figure 2**). Of note, the reversibility of these transcriptional programs and metabolic states only highlights the enormous plasticity of brown adipocytes, and whether they relate to distinct brown adipocyte subpopulations remains to be explored. In fact, “whitened” adipocytes maintain their epigenetic identity as *bona fide* brown fat cells and are primed to undergo a new “browning” cycle upon repeat cold exposure or adrenergic stimulation (37) (**Figure 2**).

While the exact mechanisms by which brown adipocytes interconvert between distinct thermogenic capacities is not fully understood, a recent study from Sun et al. described a rare subpopulation of mature brown adipocytes, found in both mice and humans, capable of regulating thermogenesis of neighboring cells (38). Marked by elevated expression of *Cyp2e1* and *Aldh1a1*, these adipocytes have altered mitochondrial morphology, lower expression of *Ucp1* and *Cidea* and, similar to BA-L adipocytes, increase in the iBAT of mice housed at thermoneutrality. Despite the low abundance, *Cyp2e1^+^*/*Aldh1a1^+^* cells are major contributors of the thermogenic capacity of the entire fat depot. Gain and loss of function studies manipulating *Aldh1a1* showed that *Ucp1* expression and OCR inversely correlate with *Aldh1a1* expression (38). Finally, the authors demonstrated that inhibition of thermogenic activity is mediated by increased production and secretion of acetate, which signals in a paracrine manner through Gpr43 (38, 39).

## Plasticity of Brown Adipocytes

Considering the capacity of brown adipocytes to adjust thermogenic output and directly regulate neighboring cells in response to environmental cues, the following questions are proposed: what are the modalities by which adipocytes are wired to dynamically respond to external signals? And what are the inputs that dictate these abilities? While it is generally appreciated that sympathetic innervation is a major contributor to the induction of the thermogenic gene program, Song et al. found that the fates of high and low thermogenic adipocytes is not due to differences in innervation (36). In mice, lineage tracing demonstrated that brown adipocyte differentiation initiates *in utero*, and that mature adipocytes persist with very low turnover throughout lifespan (36). Intriguingly, the transcriptional programming of BA-H and BA-L cells appears to occur shortly after birth, and the proportions of these cell types reach equilibrium by postnatal day 7 (P7) (36). Similarly, in humans, the number of adipocytes shows rapid increase during childhood and adolescence, but then stabilizes and remain set in adulthood in both lean and obese subjects, with an approximately 10% annual turnover (40). Alongside early-life determination of brown adipocyte developmental trajectory, thermogenic capacity appears to be in part controlled by epigenetic mechanisms. An interesting study performed by Sun & Dong et al. found that the offspring of cold-exposed male mice have elevated BAT activity and function, with improved cold tolerance, higher sympathetic innervation, increased OCR and elevated expression of classical thermogenic genes (41). Sperm derived from cold-exposed mice have marked differential methylated regions (DMRs) that enrich for neuronal development and signaling as well as metabolic processes, providing evidence that hereditary and early-life programming of brown adipose depots correlates with thermogenic functions (41). Additional studies have implicated additional epigenetic machineries in contributing to thermogenic capacity in brown adipocytes through adrenergic-dependent (42) and adrenergic-independent (43) mechanisms. Future studies to identify the signaling and epigenetic mechanisms that specifically pre-wire thermogenic capacity and mediate fate-switching will advance our understanding of brown adipose tissue function.

## Heterogeneity of Brite/Beige Adipocytes

Brown-like adipocytes possessing thermogenic capacity are found in classical white fat pads (44, 45) and have been named brite (brown-to-white) or beige adipocytes. The prevalence of these cells can vary significantly and relies on genetic factors (46), as well as physiological (34, 47), pharmacological (34, 48, 49) and pathological (50, 51) cues. Although less understood compared to their neighbor white adipocytes, brite/beige fat cells also present some degree of heterogeneity. The characteristic mark of brite/beige cells is the presence of detectable levels of UCP1 (49, 52). However, the Farmer lab showed that these cells can have distinct transcriptional signatures dependent on the browning signal that led to their recruitment/expansion (53), suggesting that beige subtypes may arise from different origins. Additionally, another subtype of beige cells (named g-beige fat) marked by enhanced glucose oxidation and derived from a distinct cellular lineage has also been identified (54). Though, not all brite/beige cells express Ucp1. In search of an answer to the fact that Ucp1 null mice gradually acclimated to cold can survive as well as wildtype mice, the Kajimura lab identified a Ucp1-independent thermogenic mechanism that relies on ATP-dependent Ca^2+^ cycling through the sarcoendoplasmic reticulum Ca^2+^-ATPase 2b (SERCA2b) and ryanodine receptor 2 (RyR2) (31), providing evidence for another, distinct subpopulation of beige adipocytes (**Figure 1**).

## Adipose Resident Immune Cells

Innate and adaptive immune cells play critical roles in the maintenance and turnover of adipose tissue. To ensure homeostasis, adipose depots undergo remodeling and constant turnover to enact necessary functions upon challenge with cold stress and nutrient depletion/enrichment, which is discussed in more detail in following sections. Regardless, immune cells play an important role in preserving the metabolic and structural flexibility of adipose tissue. Clearance of lipid-laden cells requires specialized metabolic machinery, thus adipose-resident immune cells are, relative to other non-adipose-residing immune cells, uniquely able to induce expression of the master regulator of lipid metabolism, *PPARG* (55–57). Therefore, immune cells within the adipose tissue are specialized to function within the context of the organ. However, this does not exempt them from dysfunction. As discussed in later sections, modulation of immune cell proportions, transcriptional programs and activity can have drastic consequences on both depot and systemic homeostasis. The diversity of innate and adaptive immune cells residing in the adipose tissue is substantial. An in-depth discussion of each population is beyond the scope of this review and is comprehensively covered elsewhere (58–62). Nevertheless, a brief synopsis of resident immune cells is worthy of discussion.

Recent sc-RNAseq studies (19, 63–65) have confirmed previous reports (66, 67) that macrophages (commonly referred to as adipose tissue macrophages, ATMs) are one of the dominant immune cell types by proportion and function within the adipose tissue. Transcriptional profiling of ATMs demonstrates that they also have divergent functions in specialized niches within the tissue, as reported by multiple groups (19, 64, 68–70). These include perivascular (PVM), non-perivascular (NPVM), lipid-associated (LAM) and collagen-expressing (CEM) macrophage populations, as recently denoted by Sárvári & Van Hauwaert et al. (19). Generally, macrophages strictly balance pro- and anti-inflammatory processes within the tissue to maintain a proper spatial structure. By modifying extracellular matrix, clearing debris and acting as buffers of lipid metabolism to prevent pathogenic signaling or ectopic accumulation (71), ATMs are major controlling hubs of depot dynamics. Additionally, other innate immune cell types reside within the adipose tissue (19, 63–65). Innate T cells serve as a bridge between the innate and adaptive immune responses and regulate activity of cells in both immunity classes through cytokine release. Natural killer (NK) cells, innate lymphoid (ILC) cells, and innate T cell populations including mucosal-associated invariant T (MAIT), invariant natural killer T (iNKT) and γδ T cells, have all been demonstrated to reside within adipose depots. Similarly, classical adaptive immune cells also reside within adipose depots, including CD4^+^ helper T and CD8^+^ cytotoxic T cells, along with B cells (19, 63–65). However, the density and activity of these cells is highly dependent on environmental signals and presence/absence of pathophysiology, which is discussed in more detail in later sections.

## Heterogeneity of Adipocyte Progenitors

The plasticity of the adipose tissue is the product of complex interactions between resident cell types. Through cell-cell crosstalk, cell differentiation and environmental adaptation, the adipose tissue is histologically and functionally modulated. The maintenance of a healthy adipose phenotype under post-prandial conditions and excess caloric intake is largely attributed to expansion of adipocytes through hyperplasia, rather than adipocyte hypertrophy (72). Therefore, adipocyte precursors and progenitor cells (APCs) are an integral subpopulation that has important implications for prevention and treatment of metabolic disorders. The structural and metabolic changes that fibroblast-resembling APCs undergo to form lipid-laden mature adipocytes is quite extreme. To ensure that cellular differentiation occurs properly, APCs undergo drastic transcriptional rewiring in order to adopt a mature adipocyte fate (73). Upon release from a quiescent stem-like phase, APCs rapidly modulate genes related to cell cycle, growth and protein synthesis. Following this early priming phase, the transcriptional profile switches to focus on extracellular and structural remodeling, followed by late-stage changes that activate new central metabolic networks. The dynamics of APC populations and how they contribute to the functional heterogeneity of adipose depots has revealed much about the development, maintenance and physiology of systemic metabolic homeostasis.

Due to technical difficulties of mature adipocyte characterization at the single cell level, much focus has pertained to studying the cell populations that make up the stromal vascular fraction (SVF), which includes all non-adipocyte resident cell types within the adipose depot. Deconvolution analyses of SVF cell populations have identified unique APC populations, some of which are shared or distinct between adipose depots. To separate APCs from the other resident cell types (i.e., endothelial, immune, mesothelial, and smooth muscle cells), sorting of cells by FACS or single-cell sequencing criteria based on consensus precursor-positive (such as *Pdgfra, Cd24, Cd29, Cd34, Sca1/Ly6a)* and precursor negative (such as *Lin, Cd31, Cd45)* have enabled specific enrichment of APCs for downstream analysis. Within the past few years, numerous single-cell studies have robustly identified subpopulations of APCs and have revealed that the heterogeneity of fat progenitors relates to both differentiation trajectory and differences in function.

**Figure 3:**
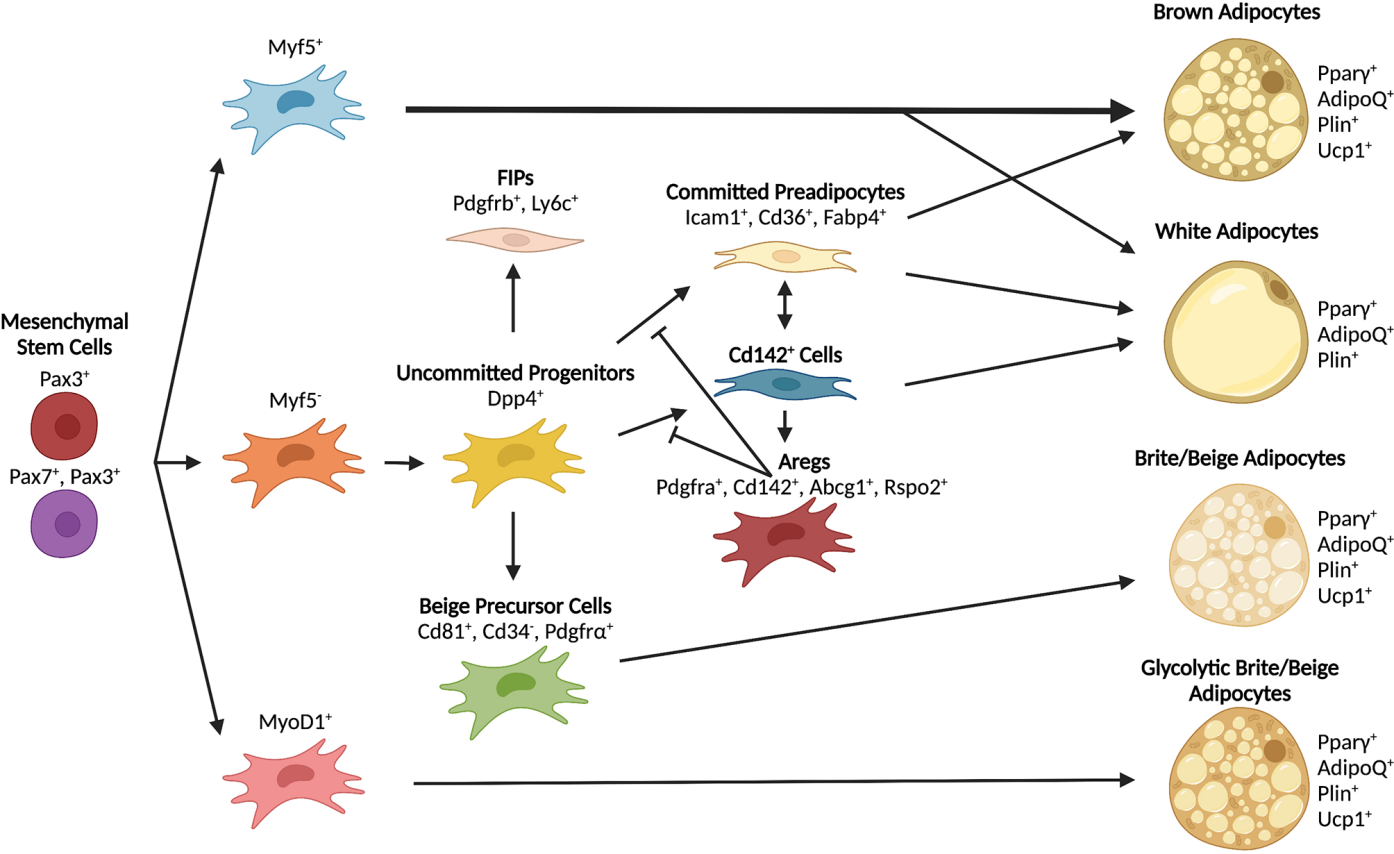
Differentiation trajectories of major adipocyte populations. Mesenchymal stem cells serve as shared early progenitors and contribute to adipocyte formation through unique lineages. The contribution of progenitor lineages varies depending on depot and adipocyte type. Some progenitors can give rise to multiple types of adipocytes depending on environmental signals and anatomical position.

Current models of adipose tissue development widely regard mesenchymal stem cells from the mesoderm as the source of embryonic precursors and progenitors of adipocytes (45, 74–77). Recent work using lineage determination of APCs has suggested that not all adipocytes derive from the same pool of cells. Lineage tracing studies in mice revealed that, while many mature adipocyte cells come from *Myf5^+^/Pax3^+^* precursors from the dermomyotome, additional precursor lineages are also involved (78). In BAT, previous studies suggested that all brown adipocytes, but not white adipocytes, arise from *Myf5^+^* progenitors (79). However, more recent work showed that not all brown adipocytes originate from a *Myf5^+^* lineage and that, in mice, *Myf5^+^/Pax3^+^* precursors also contribute to WAT development in a sex-dependent and anatomically-defined manner (78). Although it is clear that skeletal muscle and adipose share certain pools of precursors (79), the contribution of *MyoD^+^* lineages to mature adipocyte formation is controversial. Multiple studies have shown that *MyoD^+^* cells can undergo adipocyte differentiation following loss of *MyoD* expression (80–82). However, lineage tracing demonstrated that *MyoD* ^+^ precursors do not contribute to adipocyte formation *in vivo* (78). These studies indicate that both brown and white adipocytes derive from multiple lineages and contribute heterogeneously to adipocyte development *in vivo* (**Figure 3**). It remains to be confirmed whether or not the same precursor populations contribute to the development or maintenance of white and brown adipose depots in humans.

In line with the assertions that certain adipose depots may develop from distinct pools of embryonic precursors, evidence has mounted that vWAT develops from a mesothelial origin (74). Numerous follow-up characterization studies have relied on the assertion that the gene *Wt1* is specifically expressed in mesothelial cells, and that *Wt1^+^* cells are indicative of mesothelial origin (65, 74). However, a recent study found that *Wt1* is not specific to mesothelial lineage only, and that mesothelial cells do not contribute to adipocyte formation *in vivo* (83). As these results have not yet been confirmed in humans, further investigation is needed to identify whether the development of visceral depots in humans diverges from mice regarding the contribution of mesothelial progenitors to the pre- and mature adipocyte pool.

## WAT Precursors and Progenitors

The adipose tissue is constantly undergoing maintenance by controlled removal of dysfunctional cells and replacement through progenitor recruitment and differentiation. Therefore, sc-RNAseq characterizations can reveal cells at different stages of developmental trajectories. Identification of two distinct progenitor populations with divergent differentiation capacities have recently been described in mouse iWAT, with marked differences in gene expression (84, 85). In this model, TGFβ signaling serves to maintain a highly proliferative, uncommitted stem-like pool. Downstream, a second subpopulation of committed preadipocytes, derived from the former population, are primed for differentiation upon pro-adipogenic cues (84, 85). These findings largely agree with previous studies that analyzed the composition of the SVF in mouse eWAT depots under varying conditions (86, 87), in which two populations resembling stem-like progenitors with limited adipogenic potential can, under favorable conditions, progress towards a committed preadipocyte lineage. The recent pre-print from the Rosen lab generated an in-depth report of mouse adipocyte progenitor subpopulations compared across multiple studies discussed above (84–87), revealing extensive overlap consistent with previous analyses. Notably, this adipose single-cell atlas showed that scRNAseq classifications are highly consistent and confirmed the complex heterogeneity of APCs within mouse and human adipose depots (23). These studies reinforce the concept of continuous developmental trajectories that exist in defined progenitor and committed preadipocyte states.

The stem-like uncommitted progenitors identified in various studies have both shared and divergent transcriptional signatures. Burl et al. (87), Merrick & Sakers et al. (85), and more recently Rondini et al. (21) described *Dpp4^+^* cells that consist of a population of interstitial progenitors similar to other subpopulations defined by marked expression of *Ly6c1* (FIPs) (86), *Ebf2* (FAP3 and G1) (19, 84), and *Ly6a* (G4) (84). Histological analysis indicated that this population of cells lies near endothelial cells and/or within the reticular interstitium (RI) (85), a fluid-filled network containing extracellular matrix fibers, located between the endothelial and parenchymal regions of the tissue depot (88). The anatomical positioning and trajectory fate analyses suggest that this population relies on Wnt/TGFβ signaling to maintain proliferative identity. Upon appropriate environmental cues that trigger fate commitment, these cells are transcriptionally re-wired, migrate into the adipose depot as committed preadipocytes and undergo terminal adipocyte differentiation (85). This anatomical distribution was also found in human adipose tissue in which an analogous population of *DPP4^+^* cells form distinct homotypic clusters of uncommitted progenitors with adipogenic potential (20).

Similar to a consensus subpopulation of uncommitted progenitors, the existence of a defined committed lineage of preadipocytes has been reproducibly detected across multiple reports. Consistent markers for this lineage in mice include *Icam1*, *Cd36* and *Fabp4*, with slightly elevated levels of traditional adipogenic markers including *Pparg*, *AdipoQ* and *Cebpa* (19, 21, 84–87). Trajectory analysis suggests that these cells are derived from their upstream *Dpp4^+^* progenitors, with decreased proliferative capacity and robust differentiation potential in minimally permissive adipogenic conditions (84, 85). Histological evidence supports trajectory analyses, as these primed precursors are recruited out of the RI into the adipose depot to undergo differentiation (85). Therefore, a consistent body of work shows that committed preadipocytes represent an intermediate state of APCs derived from upstream progenitors recruited from surrounding perivascular and RI regions into the fat depots to complete their differentiation into mature adipocytes.

Certain APC populations are refractory to differentiation, especially uncommitted progenitors that utilize Wnt/TGFβ signaling to maintain pluripotency and proliferative capacity (19, 84–87). Interestingly, Schwalie, Dong & Zachara et al. identified in mice and humans a small subset of preadipocytes, called Aregs (adipogenesis-regulatory cells), marked by elevated expression of *Cd142* and *Abcg1*, that exert anti-adipogenic effects on committed progenitors through a paracrine mechanism (84). Numerous reports identified a similar, but larger, population of APCs expressing *Cd142* that do not possess anti-adipogenic properties (85, 86). The enrichment employed to identify the Aregs targets a specific subpopulation of *Cd142* ^+^ precursors that also express high levels of *Abcg1*, a marker not common to all *Cd142* ^+^ population (84). In fact, the expression of *Cd142* in precursor cells appears to be quite broad. Multiple groups showed *Cd142* expression across a large proportion of APCs, while *Abcg1* expression is limited only to a restricted set of *Cd142^+^* cells (85, 86). This discrepancy has since been accounted for as a product of different sorting strategies. A recent follow-up report from the Wolfrum group has confirmed that their previously identified Areg (*Cd142^+^/Abcg1^+^*) population exists as a subset of *Cd142^+^* cells within the adipose tissue. Specifically, this Areg population exerts anti-adipogenic effects on uncommitted precursors through a Wnt/β-catenin signaling cascade mediated by *Rspo2* and *Lgr4* (89) Interestingly, Aregs also express high levels of *Meox2* (84), a transcription factor that plays an important role in myogenic differentiation (80). An Areg-like population of *Cd142^+^* cells has also been reported within human and mouse skeletal muscle, which may protect against intramuscular fat deposition (90). Therefore, it has been proposed that Aregs may represent a subset of differentiation-resistant precursor cells or reflect a checkpoint state for adipogenic commitment and may either be protective or pathological depending on depot activity.

Another population of anti-adipogenic APCs functionally distinct from Aregs has also been described. *Pdgfrb^+^*/*Ly6c^+^* cells localized in visceral depots, termed fibroinflammatory progenitors (FIPs), exhibit a pro-inflammatory transcriptional profile, can potentiate the inflammatory response in macrophages, and dampen proadipogenic properties of other adipose-resident cell types (86). Notably, multiple studies reported that this cell type overlaps with *Dpp4^+^* multipotent progenitors (19, 23, 85). However, despite the similarity within transcriptional signatures, these two populations are characterized by opposite response to TGFβ. *Dpp4^+^* cells rely on TGFβ signaling to maintain their identity but, upon TGFβ signaling blockade, they can rapidly differentiate into mature adipocytes. Furthermore, *Dpp4^+^* cells efficiently differentiate into mature adipocytes when exposed to a complete adipogenic cocktail *in vitro* (85). In striking contrast, TGFβ signaling induces a pro-fibrotic phenotype in FIPs, as marked by the upregulation of collagen genes. Finally, when treated with a complete differentiation medium, FIPs display limited adipogenic capacity compared to *Dpp4* ^+^ cells (86). Further complicating the deconvolution of these populations, the Mandrup group described two fibro-adipogenic progenitor pools (FAP3 and FAP4) that partly overlap with FIPs and *Dpp4^+^* cells but do appear not to contribute to the adipocyte lineage *in vivo* (19). Therefore, it is possible that, despite the similarities of their transcriptional signatures, these three cell types may not be as comparable as suggested, and may in fact be distinct precursor subpopulations. Further work to functionally characterize *Dpp4^+^*, FIP and FAP populations will help clarify their contribution to adipose tissue development and function.

First reported in 2016, spatial transcriptomics was only recently applied to adipose tissue (20). Utilizing this platform, Bäckdahl & Franzén et al. demonstrated that cell types within the human WAT are spatially defined by a characteristic structural makeup. Human WAT architecture closely reflects the one seen in mice, with *Dpp4^+^* uncommitted progenitors forming homotypic clusters near vascular and fibrotic structures that resemble the RI. These cells were mainly found in proximity of M2-like macrophages, hinting at an immune cell-APC cross-talk that maintains APC state. Additional subpopulations of progenitors interspersed within the adipose depot also exhibited high localization scores with M2-like macrophages, which also suggests that this spatial relationship is important for adipose tissue repair and remodeling. Spatial analysis identified two major populations of mature adipocytes (Adipo^PLIN^ and Adipo^LEP^) that tend to cluster together within the depots and away from the less prominent homotypic cluster formed by a third class of adipocytes (Adipo^SAA^) (20). Collectively, this first spatial transcriptomic study of the adipose tissue revealed that human WAT consists of defined cell clustering patterns that likely represent functional spatial relationships. Future investigations into the distinct communication and interactomes of these proximally associated cell types will greatly further our understanding of cell-cell crosstalk in adipose tissue and define the dynamics associated with adipose remodeling with age and disease development. Comparison of spatial histologic features between mouse and human adipose will also be of critical importance to inform how they are functionally comparable and distinct from one another.

## Brite/Beige Precursors and Progenitors

Sensitive to cues that induce brown adipocyte expansion and activity, recruitment of beige adipocytes and trans-differentiation of resident cells can occur under cold exposure and β-adrenergic stimulation. However, beige cells are likely derived from a lineage distinct from brown adipocytes and, in some cases, also distinct from traditional white adipocytes (22). In contrast to mouse data showing that mesothelial cells do not contribute to the adipocyte pool (83), evidence exists that a subset of visceral APCs with transcriptional profiles resembling brown/beige thermogenic programs emerge from a *Myf5^-^* mesothelial origin (65). Furthermore, a recent reports from the Kajimura lab demonstrated that g-beige adipocytes can arise from a *MyoD1^+^* lineage (54), which was previously thought to not contribute to adipocyte formation in mice (78), and that a unique subset of APCs, defined by high expression of the surface markers PDGFRα, Sca1, and CD81, is specifically required for beige fat formation (91). Notably, the CD81^+^ population identified by Oguri and colleagues largely overlaps with a group of CD34^-^ APCs that were shown to give rise to Ucp1^+^ adipocytes (92). Given that white adipocytes can also undergo beigeing under appropriate environmental cues (93, 94), it seems clear that beige adipocytes arise from multiple lineages.

Intriguingly, the emergence of beige adipocytes in traditional white depots are also dependent on immunomodulatory mechanisms. Numerous reports have demonstrated that *Ucp1^+^* adipocytes and their precursors reside intimately with lymph nodes in mouse iWAT (54, 95) and human vWAT (23). In line with this localization pattern, immune cells contribute to the adoption of the thermogenic program in adipocytes (96–98). Interestingly, recent evidence emerged that the immune cell compartment of adipose tissue responds differently to cold exposure compared to traditional adrenergic stimulus (99). Whereas cold exposure preferentially promoted increased recruitment of lymphoid B and T cells, adrenergic stimulation favors recruitment of myeloid cell types such as macrophages, dendritic cells (DCs) and granulocytes *via* increased interferon/Stat1 signaling (99). Thus, the exact mechanisms by which environmental stimuli induce adipose beiging may be less similar than previously thought. Macrophages were shown to promote beiging *via* direct catecholamine production within the adipose niche (100–103). However, this concept has been questioned by other studies supporting the opposite scenario where macrophages partake in catecholamine clearance (104–106). Despite the lack of consent on what is the definitive role of macrophages in adipocyte browning/beiging, it is clear that adrenergic stimulation has a profound impact on all adipose-resident cells, not only those belonging to the adipogenic lineage, and that all cells likely contribute to the overall increase in energy expenditure. Single cell profiling of the response to β3-adrenergic receptor activators from the Granneman lab showed significant reprogramming of macrophage population, with a specific subpopulation strongly upregulating the expression of genes involved in ECM remodeling, to facilitate removal of dead adipocytes, and lipid uptake and metabolism, to support differentiation of fat cell progenitors within the adipogenic niche (87, 107). Elegant work from the Tontonoz lab also demonstrated that IL-10 represses thermogenic program in adipocytes by inhibiting recruitment of Pgc1α and C/ebpβ to thermogenic gene enhancers (108). Single cell transcriptional analysis of adipose depots pinpointed B- and T-lymphocytes as major producers of IL-10 in response to adrenergic stimulation, and selective depletion of these cell types is sufficient to replicate the phenotype observed in mice lacking IL10 or its receptor *IL10Rα* in the adipose (109). Interestingly, not all the browning effects of immune cells are linked to sympathetic nerve activation. The Czech group recently reported that the browning/beiging effects observed in mice lacking FASN specifically in the adipose is not blocked by denervation but is significantly impaired in macrophage-depleted mice (110). Although the signals involved in this immune cell-adipocyte crosstalk are yet to be identified, this work provides the first evidence of an adrenergic-independent pathway that promotes energy expenditure *via* paracrine signaling of adipose-resident cells.

If induction of beige adipocyte formation as a therapeutic approach is to be effective, selective induction, control and maintenance of these populations will be essential. Identification of transcriptional and epigenetic mechanisms of beige adipocyte formation and fate preservation implicate a number of key factors. In *MyoD1^+^* precursor cells, beige fate adoption can be controlled independently of adrenergic stimulation through activation of Gabp⍺ (54). However, there is general consensus that *Prdm16*, *Tle3*, *Ebf2* and *Zfp423* are central hubs that regulate beige adipocyte formation (111–118). To ensure recruitment of appropriate transcriptional machinery to thermogenic genes, beige adipocytes readily adopt an epigenetic landscape similar to traditional brown adipocytes (37). Upon warming, beige adipocytes readily convert back to white adipocytes, accompanied by physiological changes (unilocular lipid droplets, decreased mitochondria) and a reversion of the epigenome in line with white adipocyte gene expression (37, 94). This trans-differentiation appears to be at least partially exerted *via* increased glucocorticoid receptor activity, acting as a transcriptional activator of *Zfp423* (37). Remarkably, these whitened beige adipocytes readily convert back into thermogenic adipocytes upon repeated cold exposure (37, 94). Through maintenance of H3K4me marks at inactive enhancer and promoter elements, once-previously beige adipocytes are primed for re-activation of the thermogenic program upon repeated cold exposure (37). Additional epigenetic remodeling mechanisms have also been implicated in beige adipocyte formation, including *Tet1* (119), *Kmt5c* (42) and *Ehmt1* (43). These works demonstrate that beige adipocytes are highly plastic and rely on key epigenetic marks to maintain flexibility in response to changing environmental stimuli.

## BAT Precursors and Progenitors

Contrary to the substantial efforts put in characterizing the development of white adipocytes from APCs, deep profiling of brown APCs and their contributions to mature adipocyte populations within depots is less understood. It is well appreciated that brown adipocytes emerge from *Myf5^+^/Pax7^+^* or *Pax3^+^* multipotent stem cells that also give rise to skeletal muscle (78, 79, 81, 82). Beyond these characteristics and some understanding of transcriptional and epigenetic programs that push precursors towards a brown fate (e.g., *Prdm16*, *Ebf2*), more work remains to understand the APC pools within BAT. A recent glimpse into processes and populations controlling brown depots comes from the Seale lab, which sought to better understand adipose formation in the perivascular (PVAT) regions of both mice and humans (120). The authors found that aortic PVAT, which in mice develops between embryonic day 18 (E18) and postnatal day P3, resembles iBAT, showing multilocular adipocytes and elevated expression of thermogenic genes, including *Ucp1*. Discrete populations of fibroblast progenitors (*Pi16^+^*/*Ly6a^+^*/*Dpp4^+^*) and preadipocytes (*Pparg^+^*/*Pdgfra^+^*/*Lpl^+^*) were observed in between smooth muscle and adipocyte cells. Similar to progenitors found in WAT depots, PVAT progenitors rely on Wnt/Tgfβ signaling to maintain their progenitor status, suggesting that both BAT and WAT depots develop *via* converging signaling and cellular trajectories. Interestingly, the authors also found that, in adult mice, the predominant, postnatal PVAT preadipocyte population is lost and replaced by smooth muscle-like cells (SMC2, *Cd200^-^*/*Trpv1^+^*) with pro-adipogenic potential. Comparing mouse and human PVAT, the authors also report comforting analogies, with all major adipogenic cell types detected in mouse PVAT also found in human depots (120).

## The Impact of Obesity on the Composition of Adipose Tissue

Adipose tissue is critical for systemic insulin sensitivity and glucose homeostasis (121). Chronic insulin resistance in the adipose tissue impairs glucose uptake and prevents insulin-mediated inhibition of lipolysis, leading to excess circulating glucose and free fatty acids, leaving other tissues sensitive to lipotoxic stress vulnerable to ectopic fat accumulation and progression of metabolic dysfunction (122–124). Recent evidence from single-cell studies revealed compositional and transcriptional events occurring in visceral and subcutaneous adipose during obesity shining light on the processes that precede and contribute to adipose dysfunction and development of metabolic syndrome.

Shifts in the proportions of resident cell populations along with alterations in gene expression profiles are major contributors to pathologic depot remodeling. Recent data from single cell analysis confirms that remodeling of adipose-resident immune cells is tightly associated with changes in depot function. Although an in-depth discussion of adipose-resident immune cell population dynamics in health and diseases is summarized in detail by others (59–61, 125), an overview of adipose immune cell dynamics within the context of other resident cell types is warranted. Of all immune cell types, macrophages are the predominant class of immune cells in the adipose, and deserve to be mentioned. Obesity leads to recruitment and rapid expansion of *Trem2^+^* lipid-associated macrophages (LAMs) with increased phagocytic activity and lipid handling capacities (64, 67, 126). Notably, these cells closely associate with mature adipocytes to prevent hypertrophy and protect against adipose dysfunction (64, 68). However, in obese adipose, LAMs can also acquire proinflammatory functions by increasing the release of inflammatory cytokines, including IL-1β and TNFα (19, 63). In obesity, beyond recruitment of new macrophages, two subsets of adipose-resident macrophages called perivascular-like (PVM) and non-perivascular-like (NPVM) macrophages, normally present in lean state, also undergo profound transcriptional rewiring that boosts their lipid handling capacities similar to LAMs (19, 23, 63, 65). These data, along with trajectory analyses, further corroborate the importance of adipose-resident and newly infiltrated macrophages to the progression of adipocyte dysfunction in obesity.

**Figure 4:**
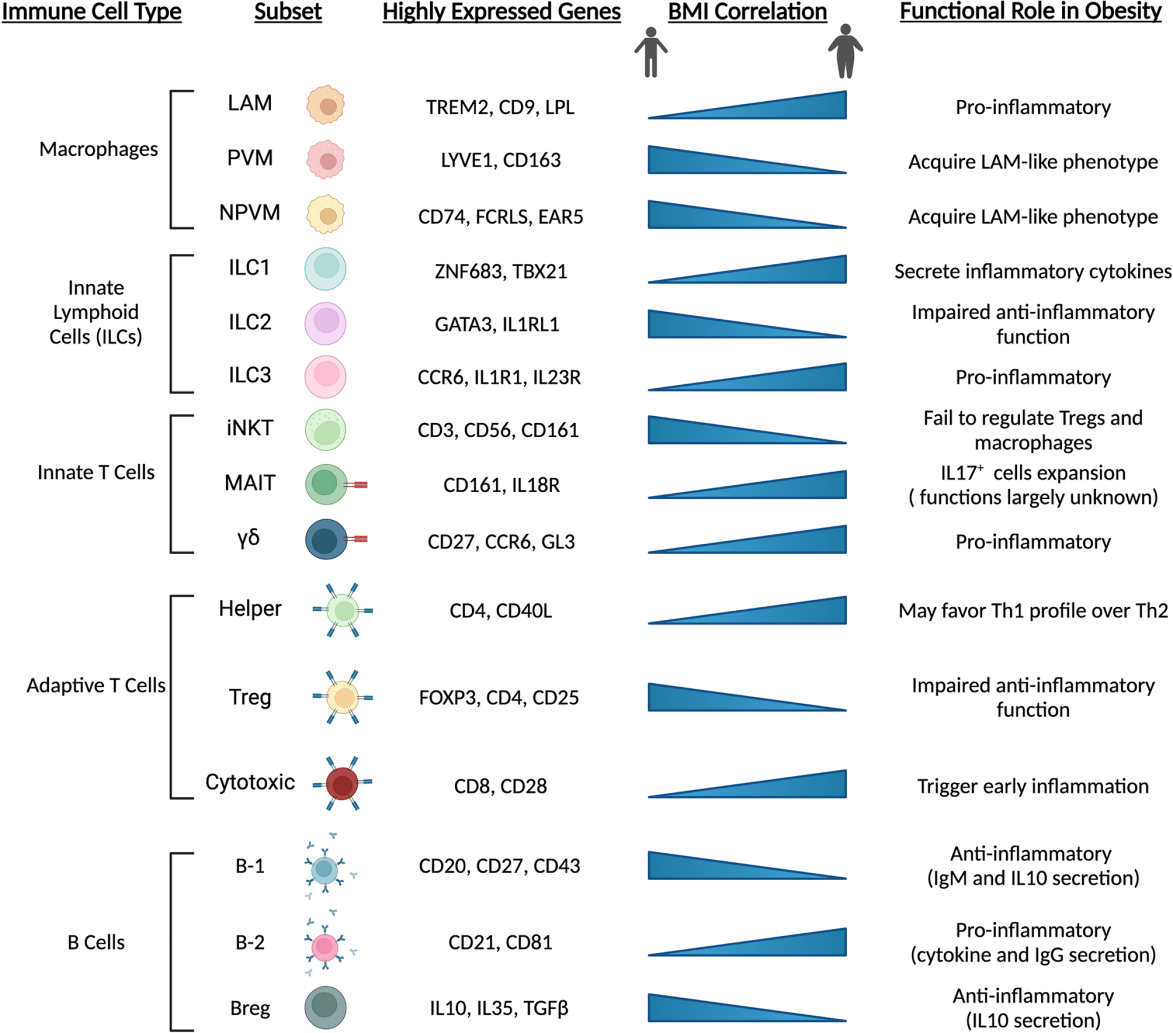
Immune cell populations are altered with obesity and high-fat diet feeding. Adipose-resident immune cells are major contributors to depot dysfunction from HFD feeding and obesity. In line with precursor and mature adipocyte populations, immune cells transition towards pro-inflammatory phenotypes and inhibit resident cell types that act to maintain an anti-inflammatory environment. Chronic activation of inflammatory immune cells exacerbates metabolic dysfunction and contributes to low-grade, chronic inflammation seen in animals and individuals with cardiometabolic disease. LAM, lipid-associated macrophage; (N)PVM, (non-)perivascular macrophage; iNKT, invariant natural killer T cell; MAIT, mucosal-associated invariant T cell; Treg, regulatory T cell; Breg, regulatory B cell.

Other innate and adaptive immune cells also appear to change during the onset of obesity and metabolic dysfunction, and further exacerbate pathophysiology (**Figure 4**). Evidence from sc-RNAseq studies corroborate previous reports suggesting numerous cell types as potential culprits in the aggravation of pathophysiology. Innate lymphoid cell (ILC) subsets undergo differential expansion or contraction during obesity. For example, ILC1 cells expand during obesity and promote tissue inflammation through inflammatory signaling, while ILC2 cells, which may function to suppress inflammation and promote Th2 phenotype of tissue-resident CD4^+^ cells, are depleted and inhibited (63, 127, 128). Additionally, ILC3 cells expand in adipose tissue with obesity and may exacerbate local tissue inflammation (63). Invariant natural killer (iNKT) cells appear to exert protective effects in response to perturbations by acting on macrophages and regulatory T (Treg) cells (129), while mucosal-associated invariant T (MAIT) and γδ T cell numbers increase and may promote an inflammatory environment in response to high-fat feeding and obesity (63, 130). CD4^+^ Treg cells act to dampen immune responses, promote an anti-inflammatory environment and are likely protective against pathologic progression, as subsets of these cells are enriched in lean mice but lost with obesity and insulin resistance (131). CD8^+^ cytotoxic T cells appear to increase in obese adipose tissue (132), and their activation is one of the earliest inflammation-inducing events occurring during the onset of obesity (133). B cells, likewise, are recruited early during the onset of obesity induced by high-fat diet (134). B-2 cells appear to promote a proinflammatory environment through release of cytokines and IgG (135), while B-1 cells and regulatory B cells (Bregs) dampen inflammation through IL10 and IgM (136, 137). Overall, obesity promotes an inflammatory environment within adipose tissue depots and exacerbates metabolic dysfunction. These pathologic changes are dampened or even reversed in animals undergoing longevity-associated lifestyle interventions such as calorie restriction (CR) and/or exercise (69, 138–141). Further elucidation of the spatial and temporal relationships of adipose-resident immune cells at the single cell level during the progression of metabolic dysfunction, and their alterations during CR or exercise regimens in the obese state may reveal promising targets for therapeutic intervention.

**Figure 5:**
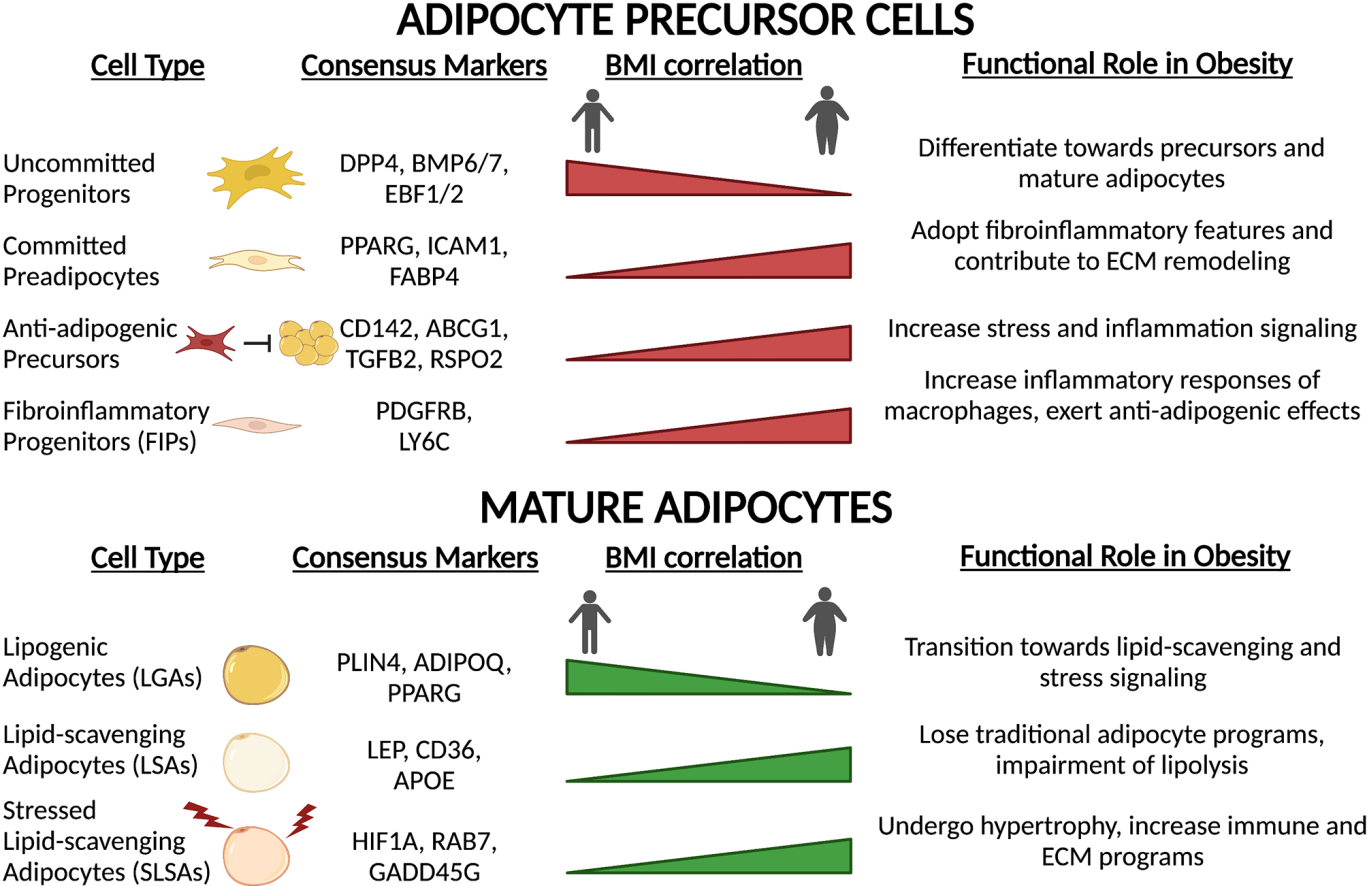
Precursor and mature adipocyte populations are altered with obesity and high-fat feeding. In response to chronic high-fat diet and obesity onset, both precursors and mature adipocytes undergo remodeling towards a chronic, low-grade inflammatory state. Transcriptional rewiring of mature adipocytes and precursors results in extensive ECM remodeling and immune signaling which contributes to adipose tissue dysfunction.

Beyond immune cells, APC populations also alter their functional capacity upon high-fat feeding, obesity onset and T2D (**Figure 5**). Transcriptional profiling of APCs and adipocytes between lean and obese states reveals a switch towards a fibro-inflammatory phenotype, indicated by elevated expression of pro-inflammatory cytokines, extracellular matrix (ECM)- and stress response-related genes (19, 23, 86, 142). The induction of collagen (*Col1a1, Col3a1*) and ECM remodeling enzymes (*Mmp1, Mmp2*) observed in obesity (19, 23, 86, 142) reflects the extensive changes to the structural environment that occur within the adipose depots under these conditions (143). Consistent with these observations, Hepler and colleagues showed an enrichment in FIPs in response to high-fat diet (86). Similarly, the prevalence of other APC subpopulations changes. Adipose tissue expansion is driven in part by adipocyte hyperplasia. To accommodate this need, multipotent *Dpp4^+^* cells decrease in abundance (19, 85), while committed *Cd142^+^* and *Icam1^+^* preadipocytes expand (19). Interestingly, these newly recruited preadipocytes show reduced lipogenic capacity due to downregulation of key lipid processing genes, a condition that further exacerbates impaired lipid handling capacity of the depot (19). In summary, high-fat diet and obesity drastically alter the composition and function of APCs promoting a pro-inflammatory phenotype, deplete early progenitor cells and increase a pool of committed preadipocytes primed for adipocyte dysfunction.

In accordance with alterations in APC function and proportions, the composition of mature adipocytes also undergoes extensive remodeling (**Figure 5**). Lipogenic adipocytes (LGAs), characterized by high sensitivity to insulin and ability to initiate *de novo* lipogenesis, make up a significant proportion of mature adipocytes in eWAT depots in lean mice (19). However, in obesity, this population decreases dramatically and is replaced by the hypertrophic expansion of lipid-scavenging (LSAs) and stressed adipocytes (SLSAs) (19). Upon high-fat feeding, both LSAs and SLSAs repress the expression of traditional adipocyte gene programs such as adipokine secretion and insulin sensitivity, and upregulate pro-inflammatory and ECM gene programs (19, 142). Additionally, lipolytic capacity is significantly hindered through decreases in central lipolysis enzyme expression (*Atgl* and *Magl)* and reduction in active Hsl (142). These observations in mice correlate with results obtained in humans. Of the three subpopulations of adipocytes identified by spatial mapping of human subcutaneous white adipose tissue (20), only one population is particularly sensitive to insulin (Adipo^PLIN^), and the proportion of this class within the fat depots negatively correlates with BMI and HOMA-IR. Temporally, these changes in adipocyte populations coincide with remodeling of macrophage populations (64), which is consistent with altered immune-adipocyte crosstalk in obese subjects (23). Taken together, these studies revealed that obesity-induced remodeling of the adipose depot is extensive and imparts negative consequences on all adipose-resident cell types: progenitors, mature adipocytes, and immune cells. Analysis of the recent single-cell resource published by Ma et al. exploring the effects of CR on age-associated changes in rodents (139) may offer insight into how health- and longevity-associated interventions may alter obesity-induced changes in the APC and adipocyte landscape of adipose tissue.

## Discussion

In the last two decades, technological advances such as CRISPR-Cas9 and scRNA-seq have significantly boosted our ability to address prominent biological questions. As scRNA-seq continues to gain in popularity, it is important to also recognize the limitations of these techniques. Extensive discussion over this topic is covered elsewhere (144–146), but a few key points warrant discussion. First, one must take into account the species of interest, biological sex and genetic backgrounds. Many similarities exist between mice and humans, but clear divergencies have also been shown, including differences in adipose deposition, sensitivity to environmental cues (diet, temperature), and even proportions of adipose resident cell populations. Second, sorting strategies that isolate cell populations of interest may further increase variability. Despite the existence of consensus markers of specific cell populations, selection strategies using known markers are intrinsically biased and are not consistent across studies, confounding results and explaining in part the differences observed between analyses. Third, the massive size of sc-RNAseq datasets requires algorithmic-based analyses to properly analyze results. QA/QC methods must be robust enough to enhance signal-to-noise ratio while maintaining high sensitivity to rare populations and low-count reads. Data analysis, oftentimes done through dimensionality reduction (PCA, UMAP), may not be consistent between studies, as there is no gold standard. However, it is reasonable to expect that in the near future many of these current limitations will be addressed through improved sensitivity and standardization of methods.

Although many questions remain to be answered, researchers are equipped with an abundance of novel tools and techniques. Expansion of -omics technologies will likely continue to gain traction amongst research groups and existing datasets, coupled with generation of new ones, will offer additional opportunities to identify previously unrecognized functions of adipose cell types beyond what is currently known. Identification of epigenomic states within adipose cell populations and integration of these data with single-cell datasets will likely provide clarity regarding the function and plasticity of cell types in response to changing environments. In particular, the combination of additional -omics platforms at single-cell resolution (147), including single-cell proteomics (148, 149) and metabolomics (150, 151) integrated with spatial resolution (152, 153), will further advance our ability to understand how resident cell types respond to inputs and interact with neighbors to dictate tissue function. Utilization of these platforms to compare adipose hierarchy between health and disease states will provide powerful assessments of the changes linked with aberrant adipose function. These tools will reveal extremely useful to better understand the beiging process in humans and tailor therapeutic strategies to promote energy expenditure *in vivo*. Altogether, our understanding of adipose biology will continue to accelerate as we look forward into the next decade, and the future looks brite/beige.

## Author Contributions

DD and AG wrote the manuscript. DD prepared the figures. All authors contributed to the article and approved the submitted version.

## Funding

This work was supported in part by the DRC at Washington University, Grant No. P30 DK020579, by ACS grant IRG-19-146-54, and by NIH/NCATS through CTSA award UL1TR002373 to the UW Institute for Clinical and Translational Research.

## Conflict of Interest

The authors declare that the research was conducted in the absence of any commercial or financial relationships that could be construed as a potential conflict of interest.

## Publisher’s Note

All claims expressed in this article are solely those of the authors and do not necessarily represent those of their affiliated organizations, or those of the publisher, the editors and the reviewers. Any product that may be evaluated in this article, or claim that may be made by its manufacturer, is not guaranteed or endorsed by the publisher.
